# Current Clinical and Pre-Clinical Imaging Approaches to Study the Cancer-Associated Immune System

**DOI:** 10.3389/fimmu.2021.716860

**Published:** 2021-09-03

**Authors:** Christopher G. Mueller, Christian Gaiddon, Aïna Venkatasamy

**Affiliations:** ^1^CNRS UPR 3572, University of Strasbourg, Immunologie-Immunopathologie-Chimie Thérapeutique, Strasbourg, France; ^2^Inserm UMR_S 1113, University of Strasbourg, Interface de Recherche Fondamentale et Appliquée en Cancérologie (IRFAC), Strasbourg, France; ^3^IHU-Strasbourg (Institut Hospitalo-Universitaire), Strasbourg, France

**Keywords:** imaging, immune cells, cancer, lymph node, tertiary lymphoid structure

## Abstract

In the light of the success and the expected growth of its arsenal, immuno-therapy may become the standard neoadjuvant procedure for many cancers in the near future. However, aspects such as the identity, organization and the activation status of the peri- and intra-tumoral immune cells would represent important elements to weigh in the decision for the appropriate treatment. While important progress in non-invasive imaging of immune cells has been made over the last decades, it falls yet short of entering the clinics, let alone becoming a standard procedure. Here, we provide an overview of the different intra-vital imaging approaches in the clinics and in pre-clinical settings and discuss their benefits and drawbacks for assessing the activity of the immune system, globally and on a cellular level. Stimulated by further research, the future is likely to see many technological advances both on signal detection and emission as well as image specificity and resolution to tackle current hurdles. We anticipate that the ability to precisely determine an immune stage of cancer will capture the attention of the oncologist and will create a change in paradigm for cancer therapy.

## Introduction

The success of immune checkpoint blockade has initiated a shift in the way we view the relationship between the immune system and cancer. The immune system is no longer seen as the underdog in an unequal duel with cancer but increasingly as a powerful system, potentially capable of fighting and even eradicating tumor cells. While formerly relatively little attention has been paid to the immune system in cancer therapy, it has now been recognized to provide precious information regarding tumor staging and to present a serious therapeutic option, in particular when amplified after releasing it from immune-checkpoint blockades. This raises the question of preserving rather than resecting the local immune tissues, especially the sentinel lymph node, where many critical immune-stimulatory and modulatory processes take place. Along the same lines, preserving and amplifying existing intra-tumoral immune cells, structured or not as tertiary lymphoid tissue may benefit patients in the long run. However, this depends on an accurate means to non-invasively estimate the activity of the immune system in order to make the right choice between resection, chemotherapy and/or immune stimulatory treatment. Over the last decade, efforts to image precisely and specifically tissue and tissue-resident single cell-types have greatly accelerated so that the capacity to accurately visualize immune cells *in vivo* becomes reality (1). These technologies have seen applications to observe lymph nodes, especially its architecture and the presence of micro-metastasis, with further clinical applications including pre-operative guidance for selected lymph node resection. Intra-vital imaging techniques are being refined for direct antitumor therapy and will provide the basis for further development towards pre-operative immune cell imaging (2). Here, we review the current approaches for *in vivo* imaging in clinical and pre-clinical settings and how these technologies and methods could pave the way to intra-vital imaging of immune cells to guide oncologists in the choice of the best treatment option.

## Lymph Node Imaging in the Clinics

The spatial resolution of standard imaging modalities does not enable the direct visualization of immune cells. However, a variety of conventional imaging techniques (e.g., lymphography, ultrasound, computed tomography, magnetic resonance imaging and positron emission tomography) may be used to detect and visualize normal and pathological human lymph nodes (**Table 1**). For instance, lymphography has been widely used to image the lymph nodes and the lymphatic system (3). This technique bears the unique ability to demonstrate changes in the internal architecture of lymph nodes (with normal nodes appearing homogeneous with a fine granularity owing to the opacification of the sinus and the non-opacification of the lymphoid follicles) especially useful in pathological nodes such as in hemopathies (e.g. lymphomas) and/or genitourinary cancers. Similarly, ultrasound (US) has been extensively used to detect superficial lymph nodes and guide needle biopsies. It provides a real-time, radiation-free, access to the lymph node substructure, visualizing micro-metastasis (0.2 - 2 mm) as well as inflammatory changes within the node, resulting from either tissue inflammation or metastatic invasion (4–6). On US, inflamed lymph nodes usually present with increased size (small axis >10 mm), a thicker cortex and increased vascular flow (using Doppler mode). Such findings are not specific and it may sometimes be difficult to differentiate inflamed nodes from metastatic ones. The use of microbubble-based contrast agent, Doppler imaging (with blood flow velocity measures of microvascular network) and elastrography (using tissue stiffness) improved the US-detection of lymph node metastasis and the distinction between malignant (harder) and benign (softer) nodes (4–6). However, US is observer-dependent, limited by the depth of tissue penetration and subject to air/bone artifacts. Presently, routine lymph node imaging relies on multimodal imaging by CT (computed tomography), PET (positron emission tomography) and/or MRI (magnetic resonance imaging), which are free from limitations imposed by the type of surrounding tissue and the depth of exploration. Validated morpho-functional criteria applied to the node (i.e., small axis > 10mm, presence of necrosis, round shape) are used to detect metastasis on imaging (8, 13). Unfortunately, all techniques reveal several limitations (**Table 1**). For instance, due to their current spatial resolution and acquisition time, CT, PET and MRI are currently insufficient to detect micro-metastasis (< 2 mm), which can be an issue for the accurate preoperative staging of cancers. Additionally, they fail to provides architectural information on the lymph nodes, and CT mostly reveals changes in nodal size. Yet, enlargement of the organ may be secondary to a metastatic invasion, inflammation and/or follicular hyperplasia (9). Therefore, evaluation of lymph node size or shape is no longer sufficient and more functional approaches are required. For instance, metastatic lymph nodes present with restricted diffusion on MRI and lower rADC (apparent diffusion coefficient ratio) than inflammatory ones (21). Additionally, [18F] Fluorodeoxyglucose ([18F] FDG) uptake on PET appears more sensitive and more specific than restricted diffusion on MRI to detect metastatic nodes (14). Similarly, dual-energy CT techniques have been used to differentiate between normal, inflammatory and metastatic lymph nodes in cervical squamous cell carcinoma (10). Unfortunately, despite constant progress, the current imaging approaches remain limited to lymph nodes and have not yet reached the cellular level.

**Table 1:** Critical assessment of the imaging modalities for intravital immune assessment.

Modalities (References)	Clinical uses	Lymphatic tissue	Single cells	Limitations	Advantages	Spatial resolution
Lymphography (3)	+	+	–	Resolution, contrast agents	LN architecture, lower cost	10-20 mm
Ultrasound (4–7)	+	+ (superficial)	+ (contrast agents)	Depth, contrast agents	Lower cost	0.2-2 mm
CT (8–12)	+	+	+ (contrast agents)	Radiation	Depth	~ 1 mm (clinical)
						Lower (preclinical)
PET/SPECT (13–20)	+	+	+ (radioisotopes contrast agents)	Radiation, higher costs	Depth, activity quantification	~ 4-5 mm (clinical)
MRI (8, 9, 14, 21–30)	+	+	+ (contrast agents)	Higher costs	DepthRadiation-free	~ 1-2 mm (clinical)
						Lower (preclinical)
PA (7, 31–35)	+	+	+ (contrast/fluorescent agents)	Clinical applications	Depth, combination with NIR/ultrasound imaging	< 5 µm
OCT (36–40)	+	-/+	–	Depth	Needle OCT	< 2 mm, depth
Fluorescence (41–48)	–	-/+	+ (fluorescent probes)	Depth, clinical use	NIR, multimodalities, theranostics	NA

NA, Not available.

## From Lymphatic Tissue to Immune Cells

Pre-clinical *in vivo* micro-imaging techniques offer superior technical possibilities compared to current human imaging modalities because of *i)* the immobility of the studied specimen (e.g., *ex vivo* lymph node, anesthetized animal), *ii)* higher spatial resolutions (e.g., special coils, higher frequency probes or higher magnetic fields) and the possible use of novel contrast-agents, not approved for clinical use. Pre-clinical multimodal imaging, especially in mice, progressed from a macroscopic lymph node-centered approach to a cellular and functionally-driven aspect of imaging. For instance, the introduction of gold-nanoparticles (AuNPs) as CT contrast agent allows the detection of T cells (11) or monocytes (12). Concerning MRI, an *ex vivo* study on a limited number of samples of normal and metastatic lymph nodes produced a series of images of the node substructure and metastatic changes that presented a good correlation with the corresponding histological slides, using very high sub-millimetric spatial resolution and longer scanning time (22). Additionally, cell-specific imaging approaches make use of contrast or tracer agents to label and track cells after their intravenous injection (23, 24). Similarly, Magnetic particle imaging (MPI) of superparamagnetic iron oxide SPIO nanoparticles are promising, with new cell-specific applications awaiting further development (25–27). Recently, large progress has been made in immuno-imaging with the development of new probes targeting endogenous immune cells for PET or SPECT (single-photon emission computed tomography) and a large toolbox of lymphocyte and myeloid cell-targeting antibodies is becoming available (15). Molecular imaging has a pivotal role to visualize immune cells, such as the different immune cells and structures in the tumor microenvironment. Several advances within PET imaging of immune checkpoint blockade (e.g., PD-L1, PD-1) and CD8 have been evaluated on animal models and are now finding their way to the clinics. For instance, site-specific immune-PET tracers (64Cu-NOTA-αPD-L1 or 68Ga-NOTA-Nb109) has been developed to image PD-L1 and PD1 (68Ga-DOTA-HACA-PD1) (16–18). Additionally, specific immune-PET tracers for endogenous CD8 imaging have also been developed (19) and one of them (89Zr-IAB22M2C) is currently undergoing a human phase I clinical trial (20). Similarly, the tumor-associated-macrophages localized in a tumor microenvironment that can represent up to 50% of a tumor mass can now be visualized using macrophage-directed radiotracers in PET (e.g., using 64 Cu-labeled polyglucose nanoparticles) and MRI (28, 29). The non-invasive detection of tumor-associated-macrophages appears promising to monitor cancer immunotherapies, especially when combined with iron oxide nanoparticles, emerging as a novel prognostic assay for more refined patient stratification and personalized therapeutic choices (30).

Alternative imaging systems have been developed. Here, the challenges are specificity, sensitivity and penetrance that preclude so-far the dominance of one particular imaging approach (**Table 1**). Optoacoustic or photoacoustic tomography (PA) is based on the generation of an acoustic wave resulting from the absorption of optical energy and combines optical contrast and high ultrasonic resolution in a single modality (31). It has a spatial resolution of less than 5 µm and higher imaging depth than US. PA combined with ultrasound proved useful to image sentinel lymph node metastasis *in vivo* in a rabbit compared to histology (7). Using multi-wavelength measurements and the hemoglobin as endogenous contrast agent, venous and arterial blood flow can be imaged. Multispectral optoacoustic tomography (MSOT) offers the possibility of simultaneously imaging cell-specific signals with high tissue penetration. Near infrared (NIR) fluorescent dyes, such as indocyanine green, widely used in angiography, can detect immune cells such as macrophages in mice (41). AuNPs have seen increased use as contrast agents for PA with the advantage of different absorption spectra and multiplex targeting moieties (32). Coupled to tumor cell-specific antibodies, they allow simultaneous detection of different tumor cells (33) and antigen delivery to dendritic cells (34). NIR excitation combined with photoacoustics efficiently tracks T cells in mouse tumor models (35). Optical coherence tomography (OCT) uses interference of light rather than the sound exploited in US to generate two-dimensional cross-sectional images. Although its resolution is higher than US, it suffers from a low (~1-2 mm) penetration depth. Small lymph nodes such as those of rodents can be entirely analyzed, whereas the analysis of the larger human lymph nodes is restricted to its periphery (36–38). A means to overcome this constraint is to minimize the imaging probe to the size of a needle allowing tissue-imaging biopsies. Thus, internal human nodal B cell follicles and germinal centers can be observed (39) making it in theory possible to detect intra-tumoral tertiary lymphoid structures. Because of lack of molecular sensitivity, there have been efforts to combine OCT with fluorescence detection systems (40).

Analogous to radioisotopes or contrast NPs, various optical tags such as fluorescent dyes can be used to observe cells *in vivo*. Fluorescence imaging is well established for superficial investigations, but at the generally-employed wavelengths (from 450 nm [ultraviolet] to 650 nm [visible]) the strong interaction between the photon and the tissue that it traverses causes image inaccuracy. Photon scattering is therefore dependent on wavelength, tissue optical properties and depth of imaging (42). Confocal and multiphoton microscopy have greatly improved image resolution, with depth reaching several hundreds of µm. Further improvements were made with selective plane illumination microscopy (light sheet) and optical clearing of tissue allowing a depth of up to 2 mm. These improvements are welcomed in research laboratories but are unlikely to see clinical pre-operative applications. More promising are fluorophores with excitation and emission spectra in the NIR wavelength range (700–900 nm) or the second NIR range (up to 1700 nm) owing to their improved depth penetration (43). The cancer-cell targeting antibody recognizing EGFR (Cetuximab) coupled to IRDye800CW allows the visualization of tumor margins and the identification of lymph nodes with micro-metastasis (44, 45). Other therapeutic antibodies are undergoing repurposing with fluorescent dyes (46). In pre-clinical models, myeloid cells were detected using dual NIR imaging with NPs coupled to anti-Gr-1 and to anti-CD11b antibodies (47). Likewise, the use of anti-CD5 and anti-CD20 antibodies, coupled to NIR emitting NPs allowed the detection of CD5+ CD20+ mantle cell lymphoma (48).

## A Fine Line Between Imaging and Therapeutics: Theranostics/Theragnostics

Before fluorescence is applied to the detection of specific immune cells *in vivo*, hurdles such as the toxicity of fluorophores and the feasibility of imaging instrumentation must be taken. In this context, photoactivated therapy for directed cancer treatment should be considered. Several NPs used for imaging present themselves intrinsic cytotoxicity by liberating - upon illumination - reactive oxygen species (PDT, Photo-Dynamic Therapy), bioactive chemicals (Photo-Activated Chemotherapy, PACT), or even heat (Photo-Thermal Therapy, PTT) (49–51). Hence, these bifunctional NPs are valuable tools to simultaneously treat and assess treatment efficacy by visualizing the tumor or the immune cells. This combined approach defines the concept of theranostics. The NPs used for theranostics are of multiple sorts that can be categorized into at least two types based on the principal constituent: either purely organic or containing one or multiple metals (such as silica, zinc, gold, or ruthenium) (52, 53). The metal present in the NPs can either function as structural support for light-emitting compounds or for bioactive molecules. It can also be exploited for its intrinsic physicochemical properties. For instance, in PDT, light-sensitive transition metals, such as ruthenium, can change their peripheral electronic content to generate reactive oxygen species after reacting with O_2_. The reactive oxygen species degrade several intracellular biological macromolecules, including DNA (54). Besides directly inducing cancer cell death by apoptosis, PDT can produce danger signals that recruit and activate cytotoxic immune cells (55). Indeed, PLGA-PEG NPs stimulate the accumulation of myeloid cells within the tumor microenvironment (56). This opens interesting opportunities to assess immune cell localization within the tumors while releasing compounds capable of recruiting and activating immune cells with antitumor activity. For PACT, bioactive ligands are liberated from the bond with the metal core to stimulate immune cells or directly kill cancer cells. Similar to the NPs that are used in diagnostics, the NPs with therapeutic properties can be functionalized for targeting specifically the tumors or infiltrating cells by using antibodies or other bioactive molecules (57). So far, only a limited number of tumors are targeted by these light-induced therapies, such as head and neck cancer, melanoma and bladder cancers. A better translation of theranostics from the pre-clinical stage to the clinic is likely to depend on the ability of NPs to be activated within the tissues. In line with the trend seen in imaging, the development of NPs for theranostics is moving toward the conception of NIR-sensitive compounds. Other innovative strategies include self-illuminating NPs that would avoid the requirement of external illumination while auto-generating light for PDT and imaging (58). Another complexing factor is the choice of the necessity of oxygen within the tumor for PDT and the choice of the target when considering the development of targeted NPs using monoclonal antibodies.

## Outlook and Challenges

The imaging of immune entities, such as different immune cells and structures in the tumor microenvironment represents an area of molecular imaging that has a pivotal role for the development of personalized and modern medicine. With the rise of immunotherapy for cancer treatment, one of the main issues on imaging is the enlargement of tumors related to the infiltration of the tumor microenvironment by immune cells that may be misinterpreted for tumor progression due to cancer growth (especially as both cases would show increased [^18^F]-FDG uptake on PET). Therefore, the non-invasive molecular imaging approaches of different immune cell subtypes would represent a change of paradigm, especially in the era of immunotherapy and personalized medicine (59). While cutting-edge non-invasive imaging modalities, coupled to contrast agents with optimized cell specificity had been restricted to pre-clinical tests for years, they are now mature and are likely to reach the routine clinic in the near future. These include specific PET radiotracers for the immune checkpoint blockade or CD8/TAM that can be of crucial help for assessing the immune status of tumors and help select the patients who have the highest probability to respond to immunotherapy. Challenges will be to control cell toxicity in a way to avoid adverse effects, while preserving the option of targeted cell death for direct tumor destruction and to create inflammation. Another issue will be to refine the measures in a way to allow the distinction between pro- versus anti-inflammatory immune cell infiltration. The direct way to tackle this difficulty would be to distinguish between cell types such as M1 versus M2 macrophages or the release of inflammatory mediators such as IFN-*γ*, TNF-α or IL-1 versus anti-inflammatory IL-10 or TGF-β. An alternative, probably technically less challenging, could be to consider not only the number but also the localization of the immune cells. Their tumor-peripheral residence is likely characteristic of an inactive immune system and an ability of the tumor to resist immune attack, while an infiltrated tumor translates to an active immune cell response. To take this further, the presence of tertiary lymphoid structures comprising a large variety of hematopoietic cells including B cells in an organized fashion would suggest a chronic inflammatory and a potent anti-tumoral activity. In this general context of questioning anti- versus pro-inflammatory immune cell activity, it is important to also consider the draining lymph node. A pro-inflammatory immune cell activity in the tumor will necessarily translate into active draining lymph nodes characterized by increased size, rich immune cell traffic and extensive vascularization. All things considered, by bringing the immune cell into the foreground of the oncologist’s attention new diagnostic and therapeutic possibilities will certainly emerge. Molecular imaging and nanomedicine will probably help to improve the cancer patient management, for improved patient stratification for a more personalized care, providing a novel axis of success that has the potential to revolutionize cancer immunotherapy.

## Author Contributions

All authors contributed to the article and approved the submitted version.

## Funding

CM acknowledges the support by grants 10-IDEX-0002, 20-SFRI-0012, 11-EQPX-022, ANR-20-CE93-0006 and ANR-19-CE14-0021-02. AV is supported by French state funds managed within the “Plan Investissements d’Avenir” and by the ANR (ANR-10-IAHU-02). CG is CNRS scientist supported by grants Ligue Contre le Cancer, Strasbourg university IDEX-excellence, ITMO Cancer (TPDTRu), ITMO Cancer (HNCARTMOL), ANR-10-IDEX-0002, ANR-20-SFRI-0012, PHC-Proteus (44234XC), Alsace Contre le Cancer, ECOS-Nord (M15PS01).

## Conflict of Interest

The authors declare that the research was conducted in the absence of any commercial or financial relationships that could be construed as a potential conflict of interest.

## Publisher’s Note

All claims expressed in this article are solely those of the authors and do not necessarily represent those of their affiliated organizations, or those of the publisher, the editors and the reviewers. Any product that may be evaluated in this article, or claim that may be made by its manufacturer, is not guaranteed or endorsed by the publisher.
